# Genome assembly and gene identification of biosurfactant-producing bacteria for environmental bioremediation

**DOI:** 10.1093/bib/bbaf631.079

**Published:** 2025-12-12

**Authors:** Tanzila Khatun, Sabrina M Elias

**Affiliations:** Department of Life Sciences, School of Environment and Life Sciences, Independent University, Bangladesh; Department of Life Sciences, School of Environment and Life Sciences, Independent University, Bangladesh; Center for Computational and Data Sciences, Independent University, Bangladesh

## Abstract

**Aim:**

The main objective of this research is to investigate and identify the presence of specific genes of a novel biosurfactant-producing *Bacillus* strain isolated from hydrocarbon rich water in the Turag River. This study was conducted by combining microbial isolation, molecular biology and bioinformatics approaches where samples were collected and enriched with diesel to isolate bacteria colonies that degrade oils.

**Methods:**

Eight-gram positive isolates were selected based on the screening of drop-collapse and oil-displacement assays. The selected strain has undergone further genome level analysis as 16s rDNA sequencing were performed to confirm that the strain belongs to *Bacillus subtilis* species. Whole genome sequencing (WGS) was carried out using illumina MiniSeq and paired sequences underwent FastQC quality control followed by assembly by SPAdes and annotation through Prokka.

**Results:**

The size was 4.9MB consisting of 4,877,988 base pairs forming a circular chromosome with 1,957 contigs, 4,848 Protein Coding Sequence (CDS), 44.76% G+C content, 55 tRNA and 1 tmRNA. The total raw reads were 519.007000k and 100% reads passed the filtering process with 0.00% low quality data whereas total bases of raw data were 75.936248M. Genomic analysis includes genome annotation, Average Nucleotide Identity (ANI), digital DNA-DNA hybridization (dDDH) and the outcome of these analyses revealed that our stain have high similarity to *Bacillus subtilis subsp. subtilis str. 168* with the OrthoANI value of 98.34% and dDDH value of 84.6%.

**Conclusion:**

The annotation of whole genome identified presence of diverse biosynthetic-gene-clusters (BGCs) including a non-ribosomal peptide synthetase (NRPS) cluster encoding surfactin with the similarity of 82% and this cluster contain *srf* operon which have genes such as *srfAA*, *srfAB* and *srfAC* which are key genes responsible for biosurfactant production in *Bacillus* species.

**References:**

1. Nayarisseri A, Singh S.K. “Genome analysis of biosurfactant producing bacterium, *Bacillus tequilensis*.” PLOS ONE 2023;18(6):e0285994.

2. Andrews S. “FastQC: a quality control tool for high throughput sequence data.” Babraham Bioinformatics 2010.

3. Luo J, Wei Y, Lyu M, Wu Z, Liu X, Luo H, Yan C. “A comprehensive review of scaffolding methods in genome assembly.” Briefings in Bioinformatics 2021;22(5):bbab033.

